# 

**Published:** 1891-07

**Authors:** 


					﻿CONTENTS.
ORIGINAL COMMUNICATIONS—
A Plea for Conservative Minor Gynecology. By R. R. Kime,
M. D., Atlanta................................... 25
Syphilitic Iritis. By Charles Dunbar Roy, A. B„ M. D., Atlanta,
Ga............................................... 267
Tuberculosis. By R. W. Stewart, M. D., Pittsburg. 26
Cancer of the Rectum. By L. L. McArthur, M. D., Chicago, Ill. 27.
One Hundred Consecutive Cases of Skin Disease. By M. B.
Hutchins, M. D., AJanta, Ga.................... 27J
SOCIETY REPORTS—	J
Allegheny County Medical Society................ 281
Gynecological and Obstetrical Society of	Baltimore. 2<l
Transactions of the Gynecological Society of Chicago.	2 ’
CORRESPONDENCE—	J
Our New York Letter.............................  3
EDITORIAL—	i
To Our Subscribers............................... 33
Koch’s Treatment—and Other Treatments............ 3d
SELECTIONS—	j|
Carcinoma Lenticulare—Cancer en Cuirasse (Velpeau).	33J
Items............................... 284,	308, 317, 318, 3|
BOOK REVIEWS....................................... 3{J
				

